# Agreement and Calibration Between FreeSurfer and Visually Quality-Controlled FSL/FAST–ALVIN Lateral Ventricle Volumetry in a Population-Based MRI Cohort

**DOI:** 10.3390/brainsci16060652

**Published:** 2026-06-20

**Authors:** Daniel Cantré, Felix Streckenbach, Sönke Langner, Thomas Beyer

**Affiliations:** Institute and Policlinic of Radiology, Paediatric Radiology and Neuroradiology, Rostock University Medical Centre, Ernst-Heydemann-Str. 6, 18057 Rostock, Germany; felix.streckenbach@med.uni-rostock.de (F.S.); soenke.langner@med.uni-rostock.de (S.L.); thomas.beyer@med.uni-rostock.de (T.B.)

**Keywords:** lateral ventricles, FreeSurfer, FSL, ALVIN, Bland–Altman analysis, calibration

## Abstract

**Background/Objectives.** Automated lateral ventricle volumetry is increasingly used in population-based neuroimaging, but correlation between methods does not establish agreement of absolute volumes. We quantified agreement and calibration between FreeSurfer and a visually quality-controlled FSL/FAST–ALVIN lateral ventricle workflow within the Study of Health in Pomerania (SHIP). **Methods.** This cross-sectional agreement-and-calibration study included 2988 SHIP participants with visually accepted FSL/FAST–ALVIN total lateral ventricle volumes; paired FreeSurfer data were available for 1913 participants. FSL/FAST–ALVIN was treated as the study reference scale rather than biological ground truth. Agreement was assessed using Pearson and Spearman correlations, Bland–Altman analysis, log-ratio agreement, Lin’s concordance correlation coefficient, and a two-way mixed-effects single-measure absolute agreement intraclass correlation coefficient. Directional calibration models predicted FSL/FAST–ALVIN volume from FreeSurfer volume and were internally validated using 2000 bootstrap resamples. **Results.** In the paired sample, volumes were almost perfectly associated (Pearson r = 0.9978; Spearman ρ = 0.9974), but FreeSurfer yielded systematically lower values (mean FreeSurfer-minus-FSL bias, −3.02 mL; 95% limits of agreement, −4.52 to −1.53 mL; geometric mean FreeSurfer/FSL ratio, 0.844). Lin’s concordance coefficient and the absolute agreement ICC were both 0.9598. Calibration was strong but workflow-specific: FSL/FAST–ALVIN volume = 2.611 + 1.0210 × FreeSurfer volume (R2 = 0.9955; optimism-corrected RMSE = 0.732 mL). **Conclusions.** FreeSurfer and visually quality-controlled FSL/FAST–ALVIN preserved participant ranking extremely well but were not directly interchangeable as absolute measurements. Cross-workflow comparisons require explicit method reporting, formal agreement analysis, and calibration to the intended measurement scale; the equation should not be used as a universal conversion formula outside comparable acquisition, segmentation, QC and software settings.

## 1. Introduction

The cerebral ventricular system is routinely assessed in neuroradiology, and lateral ventricle size remains one of the most familiar imaging markers of intracranial volume redistribution. Ventricular enlargement can accompany age-related brain volume loss, neurodegenerative disease, psychiatric disorders, or hydrocephalus, yet the interpretation of any quantitative value depends on the acquisition protocol, the segmentation definition, and the software workflow. This concern becomes especially pressing when population-based imaging data are used to derive reference ranges, or when quantitative outputs are translated into clinical neuroradiology.

The Study of Health in Pomerania (SHIP) consists of population-based cohorts of adults from Northeastern Germany; participants are selected by random sampling from official resident registration offices, stratified by sex and age [1,2]. Whole-body MRI, including brain imaging, is available for a large subset of participants, and the standardized SHIP MRI datasets have supported morphometric analyses, incidental-finding assessments, and age-related imaging research [1,2,3,4]. A recent SHIP analysis compared Evans’ Index and the Frontal and Occipital Horn Ratio with volumetric lateral ventricle data in 2988 adults from the general population [5]. That study provided epidemiological context for simple ventricular indices, but it also exposed a broader methodological issue: ventricular volumes are not method-independent quantities—they depend on how the target compartment is segmented [6,7,8,9,10,11,12,13,14].

Correlation is the statistic most frequently reported in software-comparison work, yet correlation captures association rather than agreement. Two methods may rank participants almost identically while producing systematically different absolute values. Bland and Altman accordingly argued that method comparison studies must estimate bias and limits of agreement rather than rely on correlation alone [15,16]. Lin’s concordance correlation coefficient and explicitly specified intraclass correlation coefficients add useful summary information, but they do not displace the need for direct assessment of systematic bias, proportional bias, and scale differences [17,18].

Automatic Lateral Ventricle delIneatioN (ALVIN) was developed specifically for lateral ventricle segmentation and was evaluated against established neuroanatomical segmentation tools [19]. In the SHIP ventricular workflow, FAST first segments T1-weighted images into grey matter, white matter, and cerebrospinal fluid (CSF), and ALVIN then restricts the CSF mask to the lateral ventricular compartment [19,20,21]. This focused workflow is computationally efficient and suited to large cohorts, but it defines a workflow-specific lateral ventricle measurement scale rather than a fully manual anatomical reference. FreeSurfer, by contrast, assigns ventricular labels within a broader probabilistic whole-brain anatomical segmentation framework and is widely used in large neuroimaging cohorts [22,23,24]. The distinction is important because automated volumetry can be broadly comparable to manual segmentation while still showing substantial heterogeneity across settings and structures [11].

Earlier method comparison and validation studies have shown that FreeSurfer, FSL/FIRST, FSL/FAST, ALVIN, SPM, and other automated tools differ in their segmentation definitions, reproducibility, scanner or sequence sensitivity, and compartment-specific volumetric output [6,7,8,9,10,11,12,13,14]. The present study extends this literature by focusing on lateral ventricle volume in a large population-based MRI cohort and by framing the comparison explicitly as an agreement-and-calibration problem rather than as validation against a biological reference standard.

The primary aim of this work was to quantify agreement, systematic bias, proportional bias, and calibration between FreeSurfer and visually quality-controlled FSL/FAST–ALVIN lateral ventricle volumetry in SHIP. Secondary aims were to examine whether the bias between methods depended on ventricle size, participant characteristics, visual quality control category, or FreeSurfer inferior lateral ventricle components, and to assess inter-rater agreement for visual quality control ratings in a subset of participants.

We therefore addressed four linked questions. The first three were hypothesis-driven, each with an a priori expectation, while the fourth was deliberately exploratory. We first asked whether the two workflows would rank participants consistently yet still diverge in absolute lateral ventricle volume, anticipating a very high association alongside incomplete interchangeability. We next asked in which direction any systematic difference would run, expecting FreeSurfer to yield lower volumes relative to the visually quality-controlled FSL/FAST–ALVIN reference. We then asked whether a single directional calibration equation could map FreeSurfer volumes onto that reference scale, expecting it to do so with low residual error. Finally, we explored whether the method differences that persisted after calibration were related to ventricle size, participant characteristics, visual QC category, or the FreeSurfer inferior lateral ventricle share.

## 2. Materials and Methods

### 2.1. Study Design and Population

This cross-sectional method comparison study drew on the SHIP ventricular MRI dataset. SHIP is a population-based study from Northeastern Germany that was designed to investigate common diseases and their risk factors in the general adult population [1,2]. Participants were recruited from population registries in accordance with standardized SHIP procedures, and the MRI component of the study has been described previously [3,4].

The full analysis cohort comprised 2988 participants whose FSL/FAST–ALVIN lateral ventricle volumetry passed visual quality control (QC). Visual QC entailed inspection of the spatial agreement between the generated lateral ventricle mask and the corresponding T1-weighted source images; segmentations rated as showing excellent or good visual agreement were retained, whereas those with inadequate agreement were excluded. FreeSurfer volumetric data were available for 1913 of these participants, defining the primary paired method-comparison subset, while 1075 participants lacked FreeSurfer data because the project-specific SHIP data release did not include FreeSurfer outputs for these cases. FreeSurfer results had been generated within the general SHIP processing infrastructure and were accessed for the present associated project through the regular SHIP data-request procedure; the individual-level reason for the unavailability of FreeSurfer data was not recorded in the analysis dataset. The agreement and calibration analyses were therefore confined to participants with paired FreeSurfer and FSL/FAST–ALVIN measurements, and FreeSurfer availability was reported transparently rather than assumed to be random.

**Figure 1:**
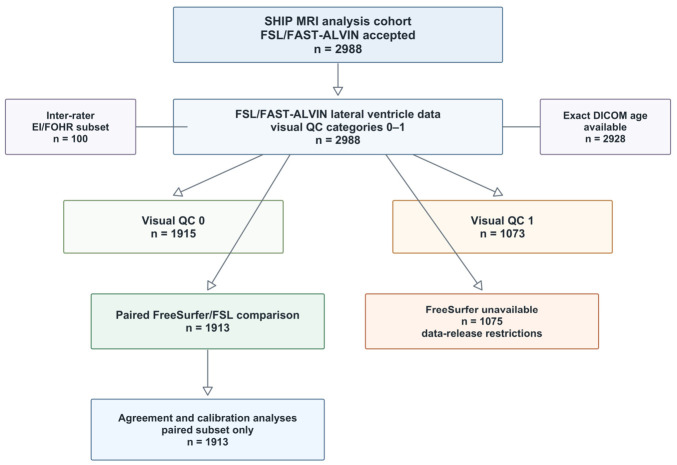
Study workflow and analysis denominators. Flowchart-style summary of the SHIP MRI analysis cohort, FSL/FAST–ALVIN availability, accepted visual QC categories, the paired FreeSurfer/FSL method-comparison subset, exact DICOM-derived age availability within the FSL/FAST–ALVIN cohort, and the inter-rater QC subset. FreeSurfer volumetry was unavailable for 1075 participants because of project-specific data-release restrictions.

Exact age at MRI was available for 2928 participants. Height and weight were captured as study variables; DICOM-derived height and weight were used solely for plausibility checks and did not enter the primary analyses. Figure 1 shows the study workflow and the analysis denominators.

### 2.2. MRI Acquisition

Brain MRI was acquired as part of the SHIP MRI protocol on a 1.5 T whole-body system (Magnetom Avanto; Siemens Healthineers, Erlangen, Germany). For the ventricular analyses, axial T1-weighted whole-brain MPRAGE images were obtained with 1 mm isotropic voxel size, repetition time 1900 ms, echo time 3.4 ms, and a flip angle of 15°.

### 2.3. Visually Quality-Controlled FSL/FAST–ALVIN Lateral Ventricle Volumetry

The target measurement scale for the present analysis was the visually quality-controlled FSL/FAST–ALVIN total lateral ventricle volume. MRI data were processed with the FMRIB Software Library (FSL, version 4.1.8). Brain extraction was performed with BET (version 2.1; fractional intensity threshold, 0.3), and the resulting brains were spatially normalized to the Montreal Neurological Institute template using FLIRT (version 5.5) and FNIRT (build 417). FAST (version 4.1) was then used to segment grey matter, white matter, and cerebrospinal fluid at the original spatial resolution [20,21].

ALVIN was applied to delineate the lateral ventricular cerebrospinal fluid compartment [19]. The ALVIN mask, which encompasses the frontal, temporal, and occipital horns of both lateral ventricles, was resampled to a 1 × 1 × 1 mm voxel grid and divided along the mid-sagittal plane to obtain separate left- and right-sided masks. Left, right, and total lateral ventricle volumes were computed in milliliters.

All lateral ventricle masks were subjected to visual QC in FSLView (version 3.1.8) by overlaying the segmentation onto the corresponding T1-weighted source image. QC category 0 denoted excellent visual agreement, QC category 1 denoted good agreement with only minor boundary mismatch or limited non-ventricular overlap, and QC category 2 denoted inadequate agreement with gross mismatch. Only segmentations in categories 0 and 1 were retained, and no manual correction of accepted masks was performed thereafter. Visual QC reduced gross segmentation error but did not transform the workflow into an anatomical ground truth.

The FSL/FAST variables for cerebrospinal fluid, grey matter, white matter, brain volume, and total intracranial volume were carried forward only as contextual or exploratory covariates. These tissue- and brain-volume variables were not separately visually quality-controlled and were not treated as equivalent to the visually controlled lateral ventricle reference scale.

### 2.4. FreeSurfer Volumetry

FreeSurfer lateral ventricle volumes obtained from the SHIP processing pipeline served as the comparator method. The outputs used here were generated using FreeSurfer version 5.1.0 and underwent the automated quality control procedures applied within SHIP. FreeSurfer provides automated anatomical segmentation and ventricular labels as part of its standard processing stream [22,23]; SHIP FreeSurfer processing and related automated segmentation work have been described previously [24].

FreeSurfer produces two lateral ventricular labels per hemisphere: the main lateral ventricle label, which includes the frontal horn, body, and occipital horn, and the inferior lateral ventricle label, corresponding primarily to the temporal horn [22,23,24]. FreeSurfer volumes were reported in mm^3^—numerically equivalent to microlitres—and were converted to milliliters by division by 1000. The primary FreeSurfer comparator was the total lateral ventricle volume, defined as the sum of the left and right main lateral ventricle labels and the left and right inferior lateral ventricle labels. The main and inferior lateral components were also analyzed separately because FSL/FAST–ALVIN does not provide a separate temporal-horn label, and this definitional difference could theoretically contribute to workflow-dependent volumetric differences.

### 2.5. Inter-Rater QC Assessment

A randomly selected inter-rater subset was assessed by two readers: a trained doctoral researcher and an experienced, board-certified neuroradiologist with more than fifteen years of neuroimaging experience. Paired visual QC ratings were available for 102 participants. These data were used to characterize the reproducibility of the QC inclusion decision, not as a manual volumetric reference standard.

### 2.6. Statistical Analysis

Each participant contributed one paired FreeSurfer and FSL/FAST–ALVIN total lateral ventricle volume to the main analyses. Statistical computations were performed in R version 4.6.0 (R Foundation for Statistical Computing, Vienna, Austria), with SPSS-format study data imported via the haven package (version 2.5.5). The full descriptive cohort comprised all 2988 participants with visually accepted FSL/FAST–ALVIN lateral ventricle volumes; the primary method comparison analyses were based on the 1913 participants with paired FSL/FAST–ALVIN and FreeSurfer measurements.

Following the four questions posed in the Introduction, the analysis proceeded from a description of the cohort and data availability to an assessment of rank-order agreement and the systematic scale difference, to directional calibration, and finally to an exploratory examination of the determinants of the method difference supported by sensitivity analyses. None of these steps were intended to establish a manual anatomical ground truth; all agreement and calibration estimates refer to the paired FreeSurfer/FSL method-comparison subset.

Continuous variables were summarised as mean ± standard deviation, median with interquartile range, and range; categorical variables were summarised as counts and percentages. Missing data were handled by complete-case analysis for each descriptive table, agreement statistic, or model. To address possible FreeSurfer-availability bias, participants with and without available FreeSurfer data were compared descriptively for age, sex, body size, visual QC category, and DICOM-derived data availability. No imputation of unavailable FreeSurfer volumes was attempted.

Agreement between methods was characterized by Pearson and Spearman correlations, Bland–Altman analysis, log-ratio agreement, Lin’s concordance correlation coefficient, and a single-measure absolute agreement ICC. The ICC was derived from a two-way mixed-effects ANOVA with participants treated as random effects and segmentation method as a fixed effect, implemented in R as ICC(A,1) using the absolute agreement formula. Pearson confidence intervals were obtained via Fisher’s z-transformation, whereas bootstrap confidence intervals based on 2000 resamples were used for Spearman correlation, the concordance correlation coefficient, and the ICC. Correlations were reported as descriptors of association rather than as evidence of interchangeability.

The signed difference was defined as FreeSurfer minus FSL/FAST–ALVIN volume, so that negative values denoted lower FreeSurfer estimates. Bland–Altman statistics included the mean bias and the 95% limits of agreement. Proportional bias was assessed by regressing the signed difference on the mean of the two methods. Because size-dependent disagreement was evident, log ratio agreement was also expressed as the geometric mean FreeSurfer/FSL ratio together with its limits of agreement.

The primary calibration model was an ordinary least-squares regression of visually quality-controlled FSL/FAST–ALVIN total lateral ventricle volume on FreeSurfer total lateral ventricle volume. This directional formulation was chosen because the practical aim was to translate FreeSurfer-derived volumes onto the FSL/FAST–ALVIN reference scale; the inverse equation was deliberately not used for back-translation. Model performance was summarised by R2, root-mean-square error (RMSE), mean absolute error (MAE), and internal validation through 2000 bootstrap resamples.

Sensitivity calibration analyses comprised a log–log model, robust regression with MASS::rlm, and equal-variance Deming regression. Deming regression was treated as an assumption-based sensitivity analysis only, because no external method-specific error-variance ratio was available.

Bias predictor models examined the log method ratio, the percentage difference, and the signed difference as outcomes. Candidate predictors comprised ventricle size, exact age at MRI, sex, body mass index (BMI), FSL/FAST total intracranial volume, FSL/FAST brain fraction, FSL/FAST–ALVIN visual QC category, and the FreeSurfer inferior lateral ventricle share. Raw total intracranial volume and raw brain volume were not entered together into the same main model to avoid unnecessary collinearity. Because size and method difference are mathematically coupled in method comparison data, the bias predictor analyses were treated as exploratory and descriptive. False-discovery-rate-adjusted *p* values were obtained with the Benjamini–Hochberg procedure.

Further sensitivity analyses involved exact age at MRI, sex- and age-stratified summaries, summaries split by visual QC category 0 versus category 1, robust calibration, and a comparison of FreeSurfer total lateral ventricle volume against the FreeSurfer main-lateral-only volume.

Inter-rater agreement for the visual QC rating was summarised by per cent agreement and Cohen’s kappa.

## 3. Results

### 3.1. Participant Characteristics and Data Availability

The analysis included 2988 participants whose FSL/FAST–ALVIN lateral ventricle segmentations passed visual QC. The mean age was 52.7 ± 13.6 years (range, 21–90 years), and 1576 (52.7%) participants were women.

FSL/FAST–ALVIN lateral ventricle volumes were available for all 2988 participants, whereas FreeSurfer volumes were available for 1913 participants (64.0%) and were absent in 1075 (36.0%) because of data release restrictions. The paired method-comparison subset had a mean age of 53.2 ± 13.3 years, comprised 1054 women (55.1%) and 859 men (44.9%), and contained 1238 segmentations in visual QC category 0 and 675 in category 1.

Compared with participants without FreeSurfer data, the paired subset was modestly older, included a higher proportion of women, had slightly lower mean BMI, and showed more complete DICOM-derived age, height, and weight availability, while the distribution of FSL/FAST–ALVIN visual QC categories was similar. Participant characteristics are summarised in Table 1, and data availability and visual QC strata are summarised separately in Table 2.

Exact DICOM-derived age at MRI was available for 2928 participants in the full dataset and for all 1913 participants in the paired analysis subset; these denominators are reported with the remaining data availability variables in Table 2.

### 3.2. Volumetric Descriptives

In the full cohort, the mean visually quality-controlled FSL/FAST–ALVIN total lateral ventricle volume was 22.82 ± 11.00 mL (median, 20.42 mL). The mean left and right FSL/FAST–ALVIN lateral ventricle volumes were 11.48 ± 5.76 mL and 11.34 ± 5.51 mL, respectively.

Within the paired FreeSurfer/FSL subset, the mean FreeSurfer total lateral ventricle volume was 19.68 ± 10.66 mL (median, 17.21 mL), and the mean left and right FreeSurfer lateral ventricle volumes were 10.27 ± 5.70 mL and 9.41 ± 5.26 mL, respectively. The FreeSurfer main lateral ventricle components accounted for the bulk of this volume (mean, 18.84 ± 10.29 mL), whereas the inferior lateral components (i.e., the temporal horns) were small in absolute terms (mean, 0.83 ± 0.53 mL; median, 0.69 mL) and contributed a mean share of 4.59 ± 2.35% (median, 4.13%) to the FreeSurfer total lateral ventricle volume. Descriptive volumetric results are provided in Table 3.

### 3.3. Agreement Between FreeSurfer and FSL/FAST–ALVIN

**Figure 2:**
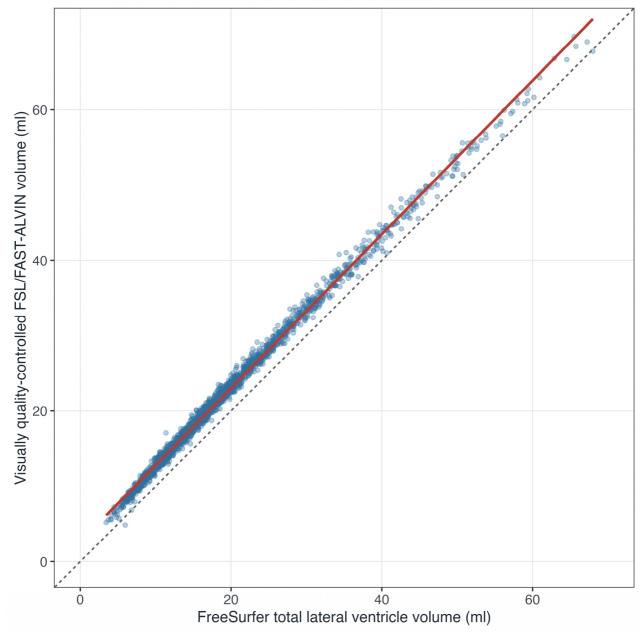
Scatterplot of FreeSurfer against visually quality-controlled FSL/FAST–ALVIN total lateral ventricle volume. The dashed line indicates identity; the solid fitted line indicates the directional linear calibration model, with the shaded band denoting the 95% confidence interval.

FreeSurfer and visually quality-controlled FSL/FAST–ALVIN total lateral ventricle volumes showed an exceptionally strong association across the paired subset (Pearson r = 0.9978, 95% CI 0.9975 to 0.9979; Spearman ρ = 0.9974, 95% bootstrap CI 0.9970 to 0.9976). This near-identical ranking of participants did not, however, imply equality of absolute measurements. Figure 2 visualizes the relationship between FreeSurfer and FSL/FAST–ALVIN total lateral ventricle volumes.

**Figure 3:**
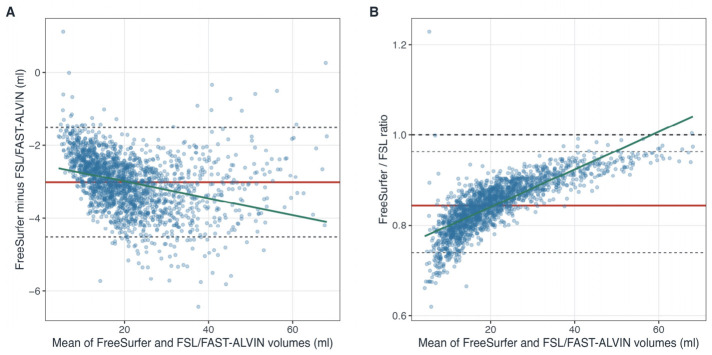
Bland–Altman and ratio agreement plots. (**A**) shows signed absolute differences against the mean of the two methods; (**B**) shows the FreeSurfer/FSL ratio against the method mean. Solid horizontal lines indicate the mean bias in (**A**) and the geometric mean ratio in (**B**); dashed lines indicate the 95% limits of agreement. The fitted trend lines visualize size-dependent bias.

Bland–Altman analysis demonstrated that FreeSurfer volumes were systematically lower than FSL/FAST–ALVIN volumes. The mean signed difference (FreeSurfer minus FSL/FAST–ALVIN) was −3.02 mL (95% CI, −3.06 to −2.99 mL), with 95% limits of agreement from −4.52 to −1.53 mL. A proportional-bias model revealed a small but statistically clear size-dependent component, with a slope of −0.0230 mL per ml of method mean (95% CI, −0.0260 to −0.0200; *p* < 0.001). Figure 3 shows the Bland–Altman and ratio agreement plots.

The ratio analysis corroborated systematic underestimation on the FreeSurfer scale. The geometric mean FreeSurfer/FSL ratio was 0.844, with ratio limits of agreement of 0.739 to 0.963. The mean percentage difference was −15.4% (limits of agreement, −26.5% to −4.4%). Lin’s concordance correlation coefficient was 0.9598 (95% bootstrap CI, 0.9561 to 0.9630), and the two-way mixed-effects single-measure absolute agreement ICC was 0.9598 (95% bootstrap CI, 0.9560 to 0.9632). Both concordance and absolute agreement estimates fell below the correlation coefficients because they penalize systematic scale displacement. Table 4 summarises the agreement statistics.

### 3.4. Calibration and Bias Predictors

The primary calibration model predicted the visually quality-controlled FSL/FAST–ALVIN total lateral ventricle volume from the FreeSurfer total lateral ventricle volume as:

*FSL*/*FAST*–*ALVIN volume* (mL) = 2.611 + 1.0210 × *FreeSurfer volume* (mL).

The calibration intercept was 2.611 mL (95% CI, 2.543 to 2.680) and the slope was 1.0210 (95% CI, 1.0179 to 1.0240). Model fit was high (R2 = 0.9955), with an RMSE of 0.731 mL and an MAE of 0.558 mL. Bootstrap internal validation yielded essentially unchanged performance estimates (optimism-corrected RMSE: 0.732 mL; optimism-corrected MAE: 0.559 mL).

**Figure 4:**
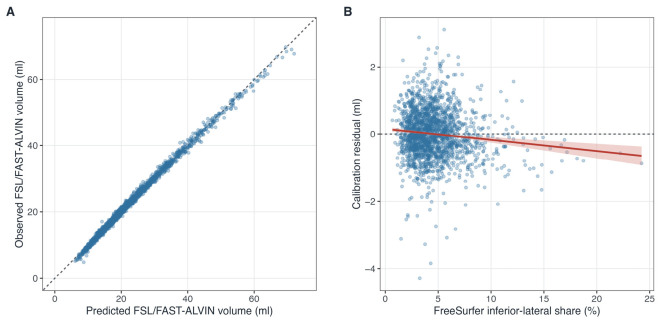
Calibration and residual assessment. (**A**) shows observed FSL/FAST–ALVIN volumes against calibrated predictions derived from FreeSurfer volumes; the dashed line indicates identity. (**B**) shows calibration residuals stratified by the FreeSurfer inferior lateral ventricle share; the horizontal dashed line marks zero residual, and the fitted line visualizes the residual trend. Dots represent individual participants; colored fitted lines indicate linear trends.

The sensitivity calibration analyses were consistent with the primary model. The log–log calibration slope was 0.8888; robust regression yielded FSL/FAST–ALVIN volume = 2.526 + 1.0252 × FreeSurfer volume; and equal-variance Deming regression—interpreted strictly as an assumption-based sensitivity analysis—yielded FSL/FAST–ALVIN volume = 2.565 + 1.0233 × FreeSurfer volume. Figure 4 displays the calibration fit and residual assessment.

Exploratory bias predictor modeling identified ventricular size as the strongest explanatory variable for the log method ratio. In the size-only model, the standardized log method mean explained a substantial fraction of the variance (adjusted R2 = 0.6837), rising to an adjusted R2 of 0.7307 once demographics, anatomy, QC category, and the FreeSurfer inferior lateral share were added. After FDR adjustment, a higher method mean, a lower total intracranial volume, a higher brain fraction, and a QC category 1 rating were all associated with a higher log ratio bias. Sex and BMI were not independently associated with the log ratio bias, and the FreeSurfer inferior lateral share ceased to be an independent predictor once the remaining covariates were accounted for (FDR-adjusted *p* = 0.701). Table 5 summarises the calibration and sensitivity calibration models; the full exploratory bias predictor coefficients are provided in Supplementary Table S2.

### 3.5. Sensitivity Analyses

The systematic negative FreeSurfer-minus-FSL bias persisted across sex, age groups, and visual QC strata.

Substituting the FreeSurfer main-lateral-only volume for the FreeSurfer total lateral ventricle volume did not improve calibration performance. The calibration based on FreeSurfer total volume achieved R2 = 0.9955 and RMSE = 0.731 mL, whereas the main-lateral-only calibration produced a lower R2 and a higher RMSE. This finding supports the use of the FreeSurfer total lateral ventricle variable as the primary comparator in the present dataset, while acknowledging that the inferior lateral components have a distinct anatomical definition. Supplementary Figure S1 displays the stratified method differences.

### 3.6. Inter-Rater QC Agreement

In the randomly selected inter-rater subset, agreement for the visual QC rating was high at 98.0%, with Cohen’s kappa = 0.954.

Variable definitions, units, and transformations are provided in Supplementary Table S1.

## 4. Discussion

This study demonstrates that an almost perfect correlation between two automated lateral ventricle volumetry workflows does not, by itself, render their absolute volumes interchangeable. Across the 1913 paired participants, FreeSurfer and visually quality-controlled FSL/FAST–ALVIN volumes ranked individuals almost identically, with Pearson and Spearman correlations both close to 0.998. Such concordance in ranking is reassuring for analyses that depend on relative ordering, but it says nothing about how comparable the volumes are in absolute terms.

The systematic scale difference between the two methods settles that question. FreeSurfer returned consistently lower volumes, with a mean FreeSurfer-minus-FSL difference of −3.02 mL and a geometric mean FreeSurfer/FSL ratio of 0.844, corresponding to volumes roughly 16% lower on the multiplicative scale. A near-perfect correlation can thus coexist with an absolute offset large enough to matter both clinically and epidemiologically, which is precisely why correlation alone cannot justify pooling volumes across workflows.

Because this offset is systematic, it is also correctable within a defined workflow. The directional model FSL/FAST–ALVIN volume = 2.611 + 1.0210 × FreeSurfer volume explained 99.55% of the variance with an optimism-corrected RMSE of 0.732 mL, and the robust and Deming sensitivity analyses produced near-identical equations. A calibration of this kind is well suited to placing FreeSurfer volumes on the visually quality-controlled FSL/FAST–ALVIN scale within a comparable processing pipeline. It should not, however, be regarded as universal: the equation is directional rather than reversible, and its use outside comparable acquisition, segmentation, QC, and software settings would require external validation.

The variation that remained after calibration could not be attributed to any single anatomical or demographic factor. Ventricle size was the strongest correlate of the log method ratio, with total intracranial volume, brain fraction, and visual QC category contributing further after adjustment, whereas sex and BMI showed no independent association. The FreeSurfer inferior lateral ventricle share, a plausible candidate given the differing label definitions, did not independently explain the log ratio bias once the remaining covariates were taken into account.

These patterns accord with well-documented differences among automated segmentation tools. ALVIN was developed specifically for lateral ventricle delineation, whereas FreeSurfer derives its ventricular labels within a broader whole-brain labeling framework [19,22,23]; differences in registration, tissue classification, partial-volume handling, label definitions, sequence or scanner context, and the treatment of inferior lateral components can each contribute to the divergence between the two volumetric scales [6,7,8,9,10,11,12,13,14].

Visual QC of the FSL/FAST–ALVIN workflow strengthened the internal reference definition but warrants careful interpretation. It reduced gross mask errors, and the inter-rater subset showed high agreement on the QC categories, yet visual QC neither produced a manual segmentation reference standard nor permitted manual correction of accepted masks. Because equivalent human review was not available for the FreeSurfer outputs, the present study estimates agreement between the available SHIP FreeSurfer workflow and a visually controlled FSL/FAST–ALVIN target workflow rather than anatomical accuracy against ground truth.

A further consideration is the availability of FreeSurfer data. Because of data-release restrictions, paired FreeSurfer volumes were available for 1913 of the 2988 participants, leaving 1075 without a FreeSurfer comparator. The paired sample remained large, but a missing fraction of this magnitude cannot simply be assumed to be random. The descriptive comparison in Table 1 and Table 2 reveals only modest differences in demographics and QC category between participants with and without FreeSurfer data; nevertheless, unmeasured selection effects cannot be excluded, and the agreement and calibration estimates should be read as specific to the paired subset.

From a clinical neuroradiological perspective, the practical implication is unambiguous: quantitative lateral ventricle volumes should not be pooled across segmentation workflows as though they were identical biological measurements. This caveat applies to normative reference work, cohort comparisons, longitudinal studies, scanner or software upgrades, and any attempt to transfer thresholds or percentiles from one workflow to another. Reports and studies should explicitly state the segmentation tool, software version, preprocessing pipeline, QC procedure, volume definition, and units; where cross-workflow comparison is unavoidable, formal agreement analysis and calibration against a pre-specified target scale become indispensable.

The inter-rater data provide a secondary quality-assurance layer. High agreement on the visual QC ratings supports the reproducibility of the inclusion decision for FSL/FAST–ALVIN segmentations. This reinforces the methodological context but does not alter the main conclusion that software-specific volumetric scales require formal agreement assessment and calibration.

## 5. Limitations

Several limitations warrant explicit consideration. First, the present work is a cross-sectional methods comparison study in a population-based cohort. No clinical endpoint, symptom, diagnosis, or outcome was used to determine whether a given ventricular volume was clinically abnormal, and the analysis therefore does not establish diagnostic thresholds for hydrocephalus, normal pressure hydrocephalus, or neurodegenerative disease.

Second, the visually quality-controlled FSL/FAST–ALVIN lateral ventricle segmentation served as the study reference scale but does not constitute a fully manual segmentation, phantom-validated measurement, or biological ground truth. The present method comparison thus quantifies agreement with this workflow-defined scale rather than absolute anatomical accuracy [11,12].

Third, FreeSurfer data were unavailable for 1075 participants because of data release restrictions. Although the paired sample remained large and Table 1 and Table 2 show the available comparison with participants lacking FreeSurfer data, selection and availability effects cannot be completely ruled out, and the calibration equation should be regarded as directly applicable only to participants with paired data.

Fourth, FreeSurfer segmentations were subjected to the automated quality control procedures of the SHIP processing pipeline, whereas FSL/FAST–ALVIN lateral ventricle masks underwent dedicated visual QC for the present analysis. This asymmetry is intrinsic to the available data and should be borne in mind when interpreting workflow-specific agreement.

Fifth, the calibration was internally validated by 2000 bootstrap resamples but not externally validated in an independent cohort, scanner, acquisition protocol, manual segmentation set, phantom dataset, or software version. External validation will be required before the equation can be applied outside the present SHIP workflow [12,13,14].

Sixth, the analysis relied on FSL version 4.1.8 and FreeSurfer version 5.1.0. Automated segmentation outputs can vary between software versions and processing configurations, so the numerical calibration reported here should not be assumed to transfer unchanged to later FreeSurfer or FSL releases or to modified pipelines [13].

Seventh, the FSL/FAST grey matter, white matter, cerebrospinal fluid, brain volume, and total intracranial volume variables were not subjected to separate visual quality control and were therefore used only as contextual or exploratory covariates rather than as visually controlled reference measurements.

Finally, the cohort comprises a population from Northeastern Germany. Generalisability to other populations, acquisition protocols, age distributions, disease-enriched cohorts, and clinical workflow settings will require further dedicated study.

## 6. Conclusions

In this large population-based SHIP MRI cohort, FreeSurfer and visually quality-controlled FSL/FAST–ALVIN lateral ventricle volumes were almost perfectly associated but systematically different on the absolute scale. FreeSurfer volumes were on average lower, and both Bland–Altman and ratio analyses confirmed that a high correlation does not amount to direct interchangeability. A simple directional calibration equation translated FreeSurfer volumes onto the FSL/FAST–ALVIN reference scale with low internal error in this workflow, but it remains acquisition-, QC-, and software-specific pending external validation. Quantitative neuroimaging studies and neuroradiological workflows should therefore report the segmentation method, software version, volume definition, QC procedure, and units explicitly, and should refrain from transferring absolute lateral ventricle thresholds between software tools in the absence of formal agreement assessment and calibration.

## Figures and Tables

**Table 1:** Participant characteristics.

Characteristic	Full Cohort	Paired FreeSurfer/FSL	FreeSurfer Unavailable
Age, years	*n* = 2988; 52.7 ± 13.6; 53.0 [43.0, 63.0]; range 21.0–90.0	*n* = 1913; 53.2 ± 13.3; 53.0 [43.0, 64.0]; range 21.0–88.0	*n* = 1075; 51.8 ± 14.1; 52.0 [42.0, 63.0]; range 21.0–90.0
Exact age at MRI, years	*n* = 2928; 53.3 ± 13.6; 53.7 [43.2, 64.0]; range 21.2–89.0	*n* = 1913; 53.7 ± 13.3; 53.8 [43.7, 64.1]; range 21.9–89.0	*n* = 1015; 52.5 ± 14.2; 52.8 [42.4, 63.7]; range 21.2–82.2
Height, cm	*n* = 2988; 169.8 ± 9.3; 170.0 [163.0, 177.0]; range 137.0–202.0	*n* = 1913; 169.4 ± 9.2; 169.0 [163.0, 176.0]; range 139.0–197.0	*n* = 1075; 170.5 ± 9.4; 170.0 [164.0, 178.0]; range 137.0–202.0
Weight, kg	*n* = 2988; 80.0 ± 15.2; 78.6 [69.0, 90.3]; range 41.5–142.7	*n* = 1913; 79.3 ± 15.1; 77.7 [68.4, 89.2]; range 41.5–135.6	*n* = 1075; 81.2 ± 15.4; 80.0 [70.4, 91.5]; range 43.6–142.7
BMI, kg/m^2^	*n* = 2988; 27.7 ± 4.5; 27.2 [24.5, 30.4]; range 17.3–48.0	*n* = 1913; 27.5 ± 4.4; 27.1 [24.4, 30.3]; range 17.3–48.0	*n* = 1075; 27.9 ± 4.6; 27.4 [24.5, 30.7]; range 17.5–43.2
Female sex	*n* = 2988; 1576 (52.7%)	*n* = 1913; 1054 (55.1%)	*n* = 1075; 522 (48.6%)
Male sex	*n* = 2988; 1412 (47.3%)	*n* = 1913; 859 (44.9%)	*n* = 1075; 553 (51.4%)

Values are shown for the full analysis cohort, for the paired FreeSurfer/FSL method-comparison subset, and for participants without available FreeSurfer volumetry. Continuous variables are summarised as mean ± standard deviation, median [interquartile range], and range. Categorical variables are shown as *n* (%).

**Table 2:** Data availability and visual quality control strata.

Characteristic	Full Cohort	Paired FreeSurfer/FSL	FreeSurfer Unavailable
FSL/FAST–ALVIN visual QC 0	*n* = 2988; 1915 (64.1%)	*n* = 1913; 1238 (64.7%)	*n* = 1075; 677 (63.0%)
FSL/FAST–ALVIN visual QC 1	*n* = 2988; 1073 (35.9%)	*n* = 1913; 675 (35.3%)	*n* = 1075; 398 (37.0%)
Exact DICOM age available	*n* = 2988; 2928 (98.0%)	*n* = 1913; 1913 (100.0%)	*n* = 1075; 1015 (94.4%)
DICOM study date available	*n* = 2988; 2928 (98.0%)	*n* = 1913; 1913 (100.0%)	*n* = 1075; 1015 (94.4%)
DICOM height available	*n* = 2988; 2907 (97.3%)	*n* = 1913; 1892 (98.9%)	*n* = 1075; 1015 (94.4%)
DICOM weight available	*n* = 2988; 2928 (98.0%)	*n* = 1913; 1913 (100.0%)	*n* = 1075; 1015 (94.4%)
FreeSurfer available	*n* = 2988; 1913 (64.0%)	*n* = 1913; 1913 (100.0%)	*n* = 1075; 0 (0.0%)

FreeSurfer data were unavailable for 1075 participants because of project-specific data-release restrictions. QC denotes visual quality control; DICOM denotes Digital Imaging and Communications in Medicine.

**Table 3:** Descriptive volumetric results. FSL/FAST–ALVIN denotes the visually quality-controlled lateral ventricle workflow based on FSL FAST and ALVIN; CSF denotes cerebrospinal fluid; TICV denotes total intracranial volume; eTIV denotes estimated total intracranial volume. The full cohort includes *n* = 2988 participants, and the paired subset includes *n* = 1913 participants. FreeSurfer volumes were converted from mm^3^ to ml by division by 1000. FSL/FAST tissue and brain-volume variables were not separately visually quality-controlled.

Workflow/Group	Variable	Unit	Analysis Set	Mean ± SD	Median [IQR]	Range
FSL/FAST–ALVIN lateral ventricles	Total lateral ventricle volume	mL	Full cohort	22.82 ± 11.00	20.42 [14.78, 27.74]	4.85–69.70
FreeSurfer lateral ventricles	Total lateral ventricle volume	mL	Paired subset	19.68 ± 10.66	17.21 [11.99, 24.20]	3.43–68.00
FSL/FAST–ALVIN lateral ventricles	Left lateral ventricle volume	mL	Full cohort	11.48 ± 5.76	10.22 [7.31, 14.10]	2.12–39.62
FreeSurfer lateral ventricles	Left lateral ventricle volume	mL	Paired subset	10.27 ± 5.70	8.99 [6.15, 12.80]	1.77–38.48
FSL/FAST–ALVIN lateral ventricles	Right lateral ventricle volume	mL	Full cohort	11.34 ± 5.51	10.10 [7.33, 13.95]	2.49–37.05
FreeSurfer lateral ventricles	Right lateral ventricle volume	mL	Paired subset	9.41 ± 5.26	8.16 [5.69, 11.48]	1.29–37.03
FreeSurfer label components	Main lateral ventricle labels	mL	Paired subset	18.84 ± 10.29	16.50 [11.43, 23.30]	2.92–65.78
FreeSurfer label components	Inferior lateral ventricle labels	mL	Paired subset	0.83 ± 0.53	0.69 [0.49, 1.00]	0.07–4.37
FreeSurfer label components	Inferior lateral share of total FreeSurfer lateral ventricle volume	%	Paired subset	4.59 ± 2.35	4.13 [3.00, 5.54]	0.67–24.24
FSL/FAST contextual tissue outputs	Cerebrospinal fluid (CSF) volume	mL	Full cohort	340.18 ± 57.49	335.65 [299.80, 375.48]	193.36–597.67
FSL/FAST contextual tissue outputs	Brain volume	mL	Full cohort	1187.49 ± 119.67	1178.65 [1100.28, 1271.60]	823.10–1803.50
FSL/FAST contextual tissue outputs	Total intracranial volume (TICV)	mL	Full cohort	1527.67 ± 143.48	1521.16 [1418.24, 1626.10]	1030.72–2199.40
FreeSurfer contextual outputs	Estimated total intracranial volume (eTIV)	mL	Paired subset	1569.93 ± 157.02	1560.00 [1460.00, 1670.00]	900.00–2320.00
FreeSurfer contextual outputs	Brain volume	mL	Paired subset	1143.05 ± 117.81	1134.56 [1058.79, 1221.44]	750.35–1777.18

**Table 4:** Agreement between FreeSurfer and visually quality-controlled FSL/FAST–ALVIN. All statistics are based on the paired method-comparison subset (*n* = 1913). The signed difference is defined as FreeSurfer minus FSL/FAST–ALVIN, so that negative values denote lower FreeSurfer volumes. Bland–Altman limits are calculated as mean bias ± 1.96 standard deviations; ratio limits are calculated on the log scale and back-transformed.

Analysis Block	Statistic	Estimate	95% CI/Note
Association	Pearson correlation	0.9978	95% CI 0.9975 to 0.9979; *p* < 0.001
Association	Spearman correlation	0.9974	95% bootstrap CI 0.9970 to 0.9976; *p* < 0.001
Side-specific association	Left-side Pearson correlation	0.9001	95% CI 0.8912 to 0.9082
Side-specific association	Right-side Pearson correlation	0.8926	95% CI 0.8831 to 0.9014
Absolute bias	Mean signed difference, FreeSurfer—FSL/FAST–ALVIN	−3.02 mL	95% CI −3.06 to −2.99 mL
Absolute bias	95% limits of agreement, FreeSurfer—FSL/FAST–ALVIN	−4.52 to −1.53 mL	Classical Bland–Altman limits
Absolute bias	Proportional bias slope, difference vs method mean	−0.0230 mL per mL	95% CI −0.0260 to −0.0200; *p* < 0.001
Ratio agreement	Geometric mean FreeSurfer/FSL ratio	0.844	Based on log(FreeSurfer/FSL)
Ratio agreement	Ratio limits of agreement	0.739 to 0.963	Back-transformed log-ratio limits
Percentage agreement	Mean percentage difference, FreeSurfer vs FSL/FAST–ALVIN	−15.4%	Descriptive mean percentage difference
Percentage agreement	Percentage-difference limits of agreement	−26.5% to −4.4%	Classical percentage limits
Concordance	Lin’s concordance correlation coefficient	0.9598	95% bootstrap CI 0.9561 to 0.9630
Concordance	Single-measure absolute agreement ICC	0.9598	95% bootstrap CI 0.9560 to 0.9632

**Table 5:** Calibration and sensitivity calibration models. All models are based on the paired method-comparison subset (*n* = 1913). The primary calibration model predicts visually quality-controlled FSL/FAST–ALVIN total lateral ventricle volume from FreeSurfer total lateral ventricle volume. OLS denotes ordinary least squares; RMSE denotes root-mean-square error; MAE denotes mean absolute error. Full exploratory bias predictor coefficients are provided in Supplementary Table S2.

Model Block	Parameter	Estimate	95% CI/Note
Primary OLS calibration: FSL/FAST–ALVIN ml from FreeSurfer ml	Intercept	2.611	95% CI 2.543 to 2.680
Primary OLS calibration: FSL/FAST–ALVIN ml from FreeSurfer ml	Slope	1.0210	95% CI 1.0179 to 1.0240
Primary OLS calibration: FSL/FAST–ALVIN ml from FreeSurfer ml	R-squared	0.9955	Apparent model fit
Primary OLS calibration: FSL/FAST–ALVIN ml from FreeSurfer ml	RMSE	0.731	Apparent error
Primary OLS calibration: FSL/FAST–ALVIN ml from FreeSurfer ml	MAE	0.558	Apparent error
Primary OLS calibration: FSL/FAST–ALVIN ml from FreeSurfer ml	Optimism-corrected RMSE	0.732	Bootstrap internal validation, 2000 resamples
Primary OLS calibration: FSL/FAST–ALVIN ml from FreeSurfer ml	Optimism-corrected MAE	0.559	Bootstrap internal validation, 2000 resamples
Sensitivity calibration	Log-log intercept	0.4865	95% CI 0.4776 to 0.4955
Sensitivity calibration	Log-log slope	0.8888	95% CI 0.8857 to 0.8919
Sensitivity calibration	Robust-regression intercept	2.526	MASS::rlm sensitivity
Sensitivity calibration	Robust-regression slope	1.0252	MASS::rlm sensitivity
Sensitivity calibration	Equal-variance Deming intercept	2.565	Assumes equal error variances in both methods
Sensitivity calibration	Equal-variance Deming slope	1.0233	Assumes equal error variances in both methods

## Data Availability

The data of the SHIP study cannot be made publicly available due to the informed consent of the study participants, but it can be accessed through a data application form available at https://fvcm.med.uni-greifswald.de/ (accessed on 16 June 2026) for researchers who meet the criteria for access to confidential data.

## Supplementary Materials

The following supporting information can be downloaded at: https://www.mdpi.com/article/10.3390/brainsci16060652/s1, Supplementary Table S1: Variable definitions, units, denominators and transformations. Supplementary Table S2: Full exploratory bias predictor model terms. Supplementary Figure S1: Distribution of FreeSurfer-minus-FSL/FAST–ALVIN differences by sex, age group and visual QC category.
