# Supplementary material for: Agreement and Calibration Between FreeSurfer and Visually Quality-Controlled FSL/FAST–ALVIN Lateral Ventricle Volumetry in a Population-Based MRI Cohort
Source: Brain Sci. 2026 Jun 20;16(6):652. doi: 10.3390/brainsci16060652 (PMC13296542; doi:10.3390/brainsci16060652)
Supplement: Supplementary file 1 [file brainsci-16-00652-s001.zip › Supplementary Table S1 Revision 2 journal corrections.pdf]

## Supplementary Table S1. Variable definitions, units, denominators and analysis roles

Definitions are synchronized with the manuscript text and table denominators.

| Manuscript variable | Source variable | Unit | Denominator | Transformation | Analysis role |
|---|---|---|---|---|---|
| FSL/FAST-ALVIN left lateral ventricle volume | fsl_alvin_latvent_left_ml | ml | n=2988 | None | Reference component |
| FSL/FAST-ALVIN right lateral ventricle volume | fsl_alvin_latvent_right_ml | ml | n=2988 | None | Reference component |
| FSL/FAST-ALVIN total lateral ventricle volume | fsl_alvin_latvent_total_ml | ml | n=2988; n=1913 paired | Left + right | Primary reference |
| FreeSurfer total lateral ventricle volume | fs_latvent_total_ul | ml | n=1913 | Divide by 1000 | Primary comparator |
| FreeSurfer main lateral ventricle volume | fs_latvent_main_total_ul | ml | n=1913 | Divide by 1000 | Sensitivity comparator |
| FreeSurfer inferior-lateral ventricle share | fs_inf_latvent_share_pct | % | n=1913 | Existing derived percentage | Bias predictor |
| Signed method difference | fs_latvent_total_ul and fsl_alvin_latvent_total_ml | ml | n=1913 | FreeSurfer ml − FSL/FAST-ALVIN ml | Agreement outcome |
| Log method ratio | fs_latvent_total_ul and fsl_alvin_latvent_total_ml | unitless | n=1913 | log(FreeSurfer ml / FSL/FAST-ALVIN ml) | Ratio agreement and bias outcome |
| Exact age at MRI | age_at_mri_exact_years | years | n=2928; n=1913 paired | Derived from DICOM dates | Sensitivity predictor |
| FSL visual QC | fsl_alvin_visual_qc | category | n=2988 | QC 0 or QC 1; QC 2 already excluded | Sensitivity predictor |
