# Supplementary material for: Agreement and Calibration Between FreeSurfer and Visually Quality-Controlled FSL/FAST–ALVIN Lateral Ventricle Volumetry in a Population-Based MRI Cohort
Source: Brain Sci. 2026 Jun 20;16(6):652. doi: 10.3390/brainsci16060652 (PMC13296542; doi:10.3390/brainsci16060652)
Supplement: Supplementary file 1 [file brainsci-16-00652-s001.zip › Supplementary Table S2 Revision 2 journal corrections.pdf]

Supplementary Table S2. Full exploratory bias-predictor model terms

Continuous predictors are standardised where indicated in the manuscript. Models are exploratory and descriptive.

Model	n	Adj. R2	Term	Estimate	SE	p	95% CI	FDR p
Size-only log-ratio bias	1913	0.684	(Intercept)	−0.1700	0.0009	<0.001	−0.1717 to −0.1683	<0.001
Size-only log-ratio bias	1913	0.684	z_mean_ml	0.0560	0.0009	<0.001	0.0543 to 0.0577	<0.001
Demographic log-ratio bias	1913	0.703	(Intercept)	−0.1616	0.0012	<0.001	−0.1639 to −0.1593	<0.001
Demographic log-ratio bias	1913	0.703	z_mean_ml	0.0610	0.0010	<0.001	0.0590 to 0.0629	<0.001
Demographic log-ratio bias	1913	0.703	z_exact_age	−0.0050	0.0010	<0.001	−0.0069 to −0.0031	<0.001
Demographic log-ratio bias	1913	0.703	sex_labelMale	−0.0186	0.0018	<0.001	−0.0221 to −0.0151	<0.001
Demographic log-ratio bias	1913	0.703	z_bmi	−0.0007	0.0009	0.403	−0.0024 to 0.0010	0.458
Anatomy/QC log-ratio bias	1913	0.731	(Intercept)	−0.1648	0.0015	<0.001	−0.1677 to −0.1619	<0.001
Anatomy/QC log-ratio bias	1913	0.731	z_mean_ml	0.0695	0.0012	<0.001	0.0671 to 0.0719	<0.001
Anatomy/QC log-ratio bias	1913	0.731	z_exact_age	−0.0039	0.0012	0.001	−0.0063 to −0.0015	0.002
Anatomy/QC log-ratio bias	1913	0.731	sex_labelMale	−0.0016	0.0023	0.472	−0.0060 to 0.0028	0.521
Anatomy/QC log-ratio bias	1913	0.731	z_bmi	−0.0009	0.0008	0.277	−0.0025 to 0.0007	0.343
Anatomy/QC log-ratio bias	1913	0.731	z_ticv	−0.0144	0.0012	<0.001	−0.0167 to −0.0120	<0.001
Anatomy/QC log-ratio bias	1913	0.731	z_brain_fraction	0.0058	0.0014	<0.001	0.0030 to 0.0086	<0.001
Anatomy/QC log-ratio bias	1913	0.731	qc_labelQC 1	−0.0125	0.0019	<0.001	−0.0162 to −0.0089	<0.001
Inferior-lateral share log-ratio bias	1913	0.731	(Intercept)	−0.1649	0.0015	<0.001	−0.1678 to −0.1620	<0.001
Inferior-lateral share log-ratio bias	1913	0.731	z_mean_ml	0.0693	0.0013	<0.001	0.0667 to 0.0719	<0.001
Inferior-lateral share log-ratio bias	1913	0.731	z_exact_age	−0.0039	0.0012	0.002	−0.0063 to −0.0015	0.002
Inferior-lateral share log-ratio bias	1913	0.731	sex_labelMale	−0.0015	0.0023	0.506	−0.0060 to 0.0029	0.531
Inferior-lateral share log-ratio bias	1913	0.731	z_bmi	−0.0009	0.0008	0.277	−0.0025 to 0.0007	0.343
Inferior-lateral share log-ratio bias	1913	0.731	z_ticv	−0.0144	0.0012	<0.001	−0.0167 to −0.0120	<0.001
Inferior-lateral share log-ratio bias	1913	0.731	z_brain_fraction	0.0058	0.0015	<0.001	0.0029 to 0.0086	<0.001
Inferior-lateral share log-ratio bias	1913	0.731	qc_labelQC 1	−0.0125	0.0019	<0.001	−0.0161 to −0.0088	<0.001
Inferior-lateral share log-ratio bias	1913	0.731	z_inf_share	−0.0003	0.0009	0.701	−0.0021 to 0.0014	0.701
Signed-difference bias	1913	0.305	(Intercept)	−2.9462	0.0271	<0.001	−2.9993 to −2.8932	<0.001
Signed-difference bias	1913	0.305	z_mean_ml	−0.0699	0.0240	0.004	−0.1170 to −0.0228	0.005
Signed-difference bias	1913	0.305	z_exact_age	−0.1209	0.0224	<0.001	−0.1649 to −0.0770	<0.001
Signed-difference bias	1913	0.305	sex_labelMale	−0.1199	0.0412	0.004	−0.2008 to −0.0391	0.005
Signed-difference bias	1913	0.305	z_bmi	−0.0104	0.0150	0.488	−0.0398 to 0.0190	0.526
Signed-difference bias	1913	0.305	z_ticv	−0.2513	0.0216	<0.001	−0.2937 to −0.2089	<0.001
Signed-difference bias	1913	0.305	z_brain_fraction	0.0746	0.0264	0.005	0.0229 to 0.1263	0.006
Signed-difference bias	1913	0.305	qc_labelQC 1	−0.0673	0.0338	0.047	−0.1336 to −0.0010	0.061
Signed-difference bias	1913	0.305	z_inf_share	0.0805	0.0163	<0.001	0.0485 to 0.1125	<0.001
Percentage-difference bias	1913	0.730	(Intercept)	−15.0272	0.1247	<0.001	−15.2717 to −14.7826	<0.001
Percentage-difference bias	1913	0.730	z_mean_ml	5.7950	0.1107	<0.001	5.5779 to 6.0121	<0.001
Percentage-difference bias	1913	0.730	z_exact_age	−0.3397	0.1032	0.001	−0.5422 to −0.1373	0.002
Percentage-difference bias	1913	0.730	sex_labelMale	−0.1864	0.1899	0.326	−0.5590 to 0.1861	0.381
Percentage-difference bias	1913	0.730	z_bmi	−0.0728	0.0691	0.292	−0.2083 to 0.0627	0.350
Percentage-difference bias	1913	0.730	z_ticv	−1.1797	0.0996	<0.001	−1.3751 to −0.9844	<0.001
Percentage-difference bias	1913	0.730	z_brain_fraction	0.4633	0.1215	<0.001	0.2250 to 0.7016	<0.001
Percentage-difference bias	1913	0.730	qc_labelQC 1	−0.9366	0.1558	<0.001	−1.2421 to −0.6311	<0.001
Percentage-difference bias	1913	0.730	z_inf_share	0.0352	0.0752	0.639	−0.1122 to 0.1827	0.655
