# Supplementary figures and images for: Agreement and Calibration Between FreeSurfer and Visually Quality-Controlled FSL/FAST–ALVIN Lateral Ventricle Volumetry in a Population-Based MRI Cohort
Source: Brain Sci. 2026 Jun 20;16(6):652. doi: 10.3390/brainsci16060652 (PMC13296542; doi:10.3390/brainsci16060652)

**A**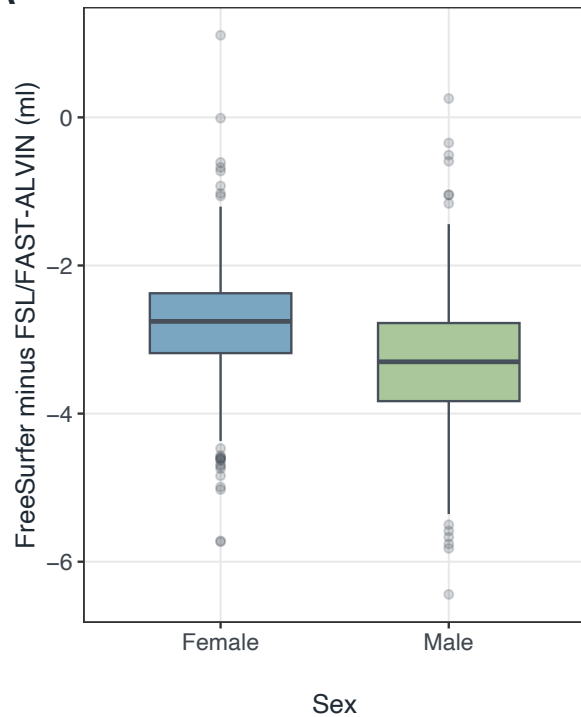**B**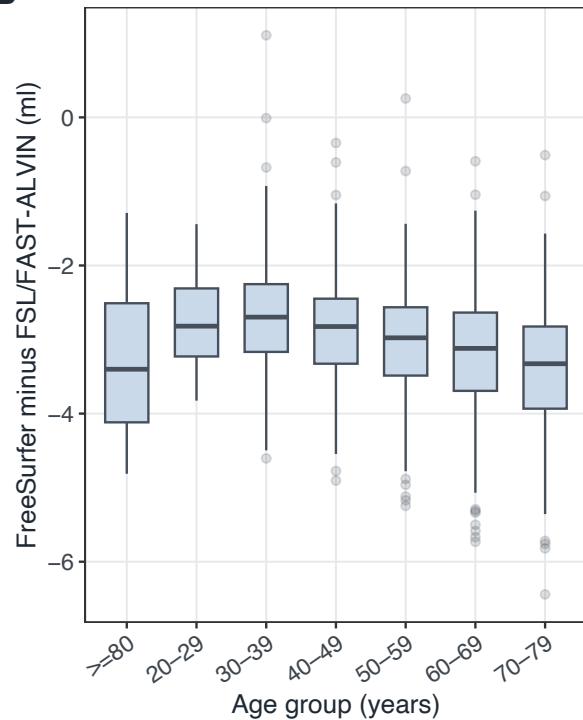**C**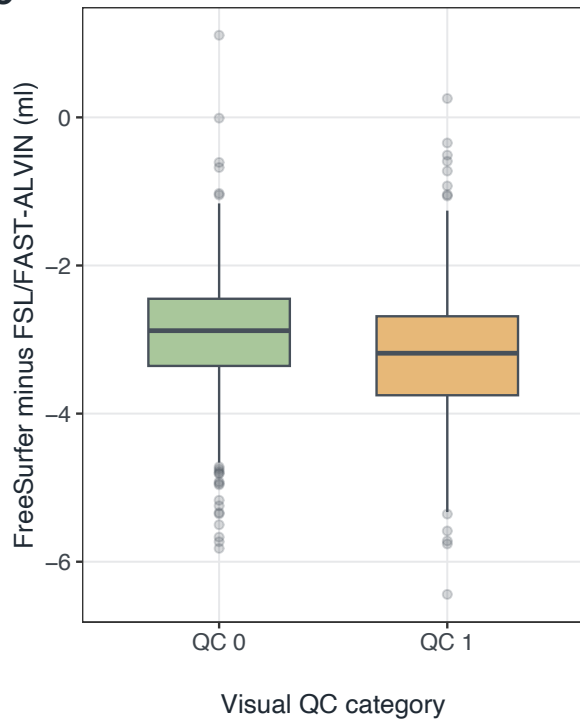

Supplement: Supplementary file 1 [file brainsci-16-00652-s001.zip › Supplementary Figure S1 Revision 2 journal corrections.pdf]

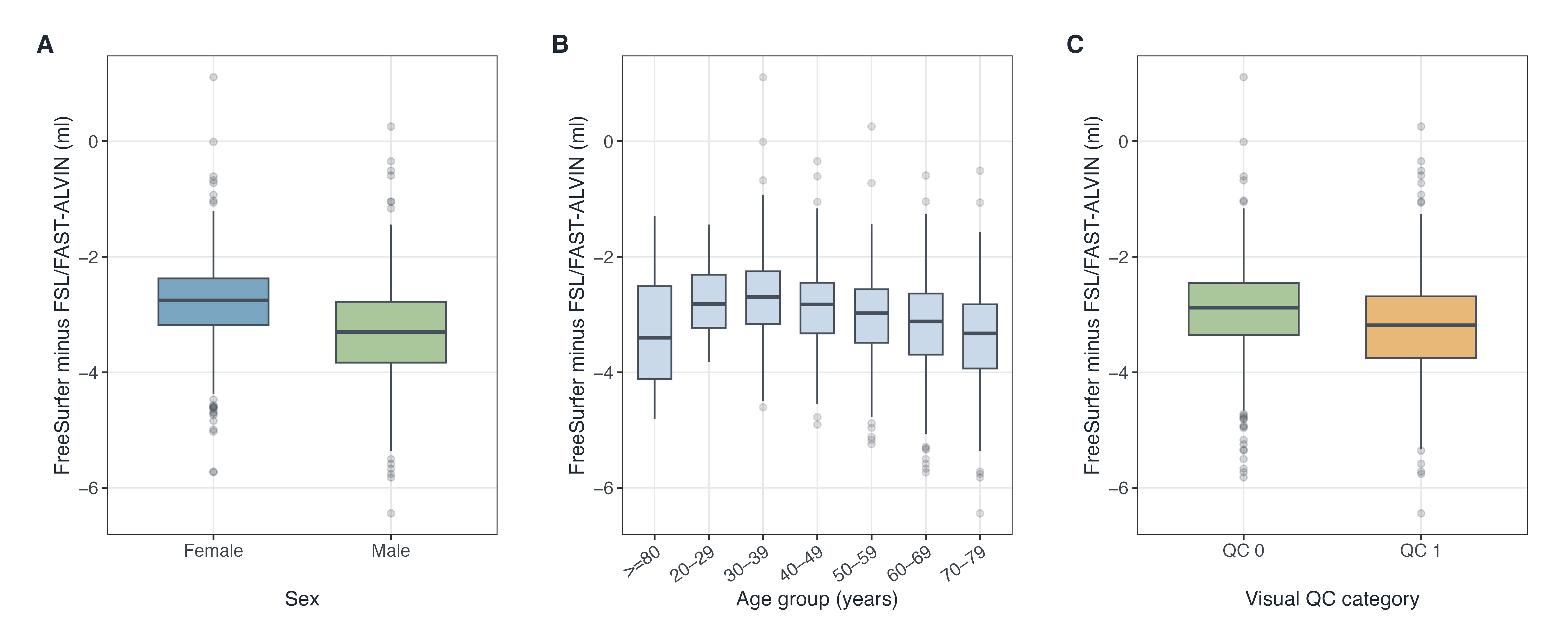

Supplement: Supplementary file 1 [file brainsci-16-00652-s001.zip › Supplementary Figure S1 Revision 2 journal corrections.png]
